# Functional Connectivity Within the Central Autonomic Network Increases During Resonance Paced Breathing at 0.1 Hz

**DOI:** 10.1111/psyp.70263

**Published:** 2026-02-23

**Authors:** M. E. Bates, L. M. Lesnewich, A. P. Pawlak, J. F. Buckman, S. Gohel

**Affiliations:** ^1^ Rutgers University New Brunswick New Brunswick New Jersey USA; ^2^ War Related Illness and Injury Study Center US Department of Veterans Affairs East Orange New Jersey USA; ^3^ Rutgers University Newark Newark, New Jersey USA

**Keywords:** functional brain connectivity, insula, resonance, respiratory intervention, slow breathing

## Abstract

Physiological processes such as respiration, while typically automatic, can be brought under volitional control to initiate cascading effects across the autonomic and central nervous systems. Slow breathing paced at approximately six breaths‐per‐minute, termed resonance paced breathing (RPB), activates the 0.1 Hz resonance frequency of the baroreflex. This slow breathing intervention has multiple health benefits, including significant reductions in anxiety, depression, and stress. Positive cardiovascular changes (e.g., reduced blood pressure, increased heart rate variability and baroreflex sensitivity) are observed during RPB, however, little is known about real‐time brain response to RPB. This study examined changes in functional connectivity between 13 a priori selected regions‐of‐interest (ROIs) of the central autonomic brain network during RPB compared to natural breathing. Participants (*N* = 147) with depression, a substance use disorder, or no diagnosis were imaged using a 3 T Siemens Trio scanner during brief episodes of natural breathing and RPB. Linear mixed modeling with adaptive false discovery rate correction indicated that compared to natural breathing, RPB led to increased functional connectivity between multiple ROIs of the central autonomic network. Of the 15 ROI pairs with significantly increased connectivity, 10 pairs involved a subdivision of the insula. This pattern supports that the insular cortex may play a key role in integrating afferent viscerosensory information from lower brain areas with cognitive and emotional information relayed from higher cortical areas. Further knowledge about the interdependencies between autonomic and central nervous system processes is important to identify new and accessible intervention targets for physical and mental disorders that involve arousal modulation challenges.

## Impact Statement

This is the first report to examine functional connectivity changes in the brain during resonance paced breathing, a respiratory intervention with empirically supported physical and mental benefits. Compared to natural breathing rates, a breathing rate of six breaths per minute increased the connectivity of brain regions involved in central autonomic nervous system control, cognition, and emotion regulation, supporting its potential as a just‐in‐time intervention to modulate arousal states in daily life.

## 1 Introduction

Interest has grown in better understanding how autonomic nervous system processes contribute to the experience and regulation of cognitive and emotional states (Quadt et al. 2022). This interest has been motivated in part by the observation that processes such as respiration, while typically operating in an automatic fashion and outside of awareness, also can be brought under volitional control to initiate cascading effects across the autonomic and central nervous systems. For example, heart rate variability (HRV) biofeedback and other respiratory interventions that involve the slow, rhythmical pacing of breath can be used to positively manipulate cardiovascular processes and outcomes (Burlacu et al. 2021). Such interventions also have demonstrated positive mental health outcomes, including medium‐to‐large effect size reductions in anxiety and stress (Goessl et al. 2017). These and other positive physical and mental health benefits of slow, paced breathing interventions (Fournie et al. 2021; Lehrer et al. 2020; Leyro et al. 2019; Reneau 2020) point to interdependencies between autonomic and central nervous system information processing. Furthering knowledge about the nature of these interdependencies is important for identifying new, accessible, and scalable interventions for disorders that involve difficulties in modulating arousal such as anxiety, depression, sleep disorders, substance use disorders, and other biobehavioral conditions (Goheen et al. 2023).

Within the Research Domain Criteria (RDoC) framework, the arousal/regulatory system is a major domain of human neurobiological functioning believed to contribute to the continuum of mental health and illness (Cuthbert 2022). Arousal is defined as a continuum of sensitivity to external and internal stimuli that operates in a context‐specific manner and is regulated by homeostatic factors (Kozak and Cuthbert 2016). This definition encompasses neural representations of arousal, subjective experiences of arousal state, and physiological arousal evident across the cardiovascular and other organ systems (Bates et al. 2022). Arousal is a key component of affective and emotional states that are common features of mental health disorders. Cardiovascular processes, along with neural processes, can be used to help quantify neurobiological arousal, its contribution to the experience of anxiety, stress, and other cognitive emotional states, and the effects of interventions that seek to modulate arousal.

A slow breathing rate of about six breaths‐per‐minute, also referred to as resonance paced breathing (RPB), is equivalent to 0.1 Hz, a resonance frequency of the baroreflex (Lehrer et al. 2000). The arterial baroreflex is a bi‐directional signaling loop that provides a continual flow of neural inputs to the heart, and afferent sensory inputs to the brain, to control moment‐to‐moment fluctuations of blood pressure caused by factors such as changes in posture, physical activity, and emotional experiences (Benarroch 2008). This feedback loop modulates arousal predictively to anticipate and prepare an organism for behavioral activation, as well as to carry out specific goal‐directed behaviors (Benarroch 1993; Gianaros and Jennings 2018; Quadt et al. 2022; Steffen et al. 2022). RPB exponentially and nearly instantaneously amplifies heart rate (HR) oscillations by synchronizing two primary influences on cardiac dynamics: vagally‐mediated respiratory sinus arrhythmia and the inherent 0.1 Hz cycle of the arterial baroreflex (Fonoberova et al. 2014; Lehrer et al. 2000). These large oscillations in HR are accompanied by vasodilation, clinically significant reductions in blood pressure, and increased baroreflex sensitivity (Lehrer et al. 2003; Vaschillo et al. 1998). Thus, RPB instigates functional enhancement of multiple cardiovascular processes, thereby amplifying afferent signaling from the cardiovascular system to the brain. The present study examined whether these amplified cardiovascular oscillations are accompanied by increased coordination of brain regions within the circuitry that mediates cardiovascular regulation via the baroreflex loop. This neural circuitry is referred to as the central autonomic network (CAN).

The neurophysiology, functional anatomy, and molecular mechanisms of the CAN have been extensively characterized in preclinical rodent and primate models (Benarroch 1993, 2008; Goldstein 2001). CAN anatomy also has been demonstrated using data from human neuroimaging studies (Beissner et al. 2013; Cechetto and Shoemaker 2009). Information from cortical, subcortical, and brain stem structures projects to the heart at the sinoatrial node by the sympathetic and parasympathetic branches of the autonomic nervous system. Information from the heart and blood vessels is conveyed to the brain via baroreceptors located mainly in the walls of the aorta and carotid artery. This afferent signaling path enters the brain via the nucleus of the solitary tract in the brain stem and then progresses through midbrain relay stations. Ultimately, the pathway integrates with other sensory, cognitive, and affective information as it ascends to cortical regions, including the medial frontal, cingulate, and insular cortices. When afferent signals to this network are strongly modulated, as occurs during RPB and HRV biofeedback, it seems reasonable to expect upstream effects on the coordination of brain region activation within the CAN.

Indeed, it has been hypothesized that respiratory interventions that increase the amplitude of heart rate oscillations may lead to increased coordination within brain networks that share circuitry with the CAN (Mather and Thayer 2018; Nashiro et al. 2023). While, to our knowledge, there are no prior studies of changes in brain network coordination during RPB, a few studies have examined how a several‐week course of HRV biofeedback may result in chronic changes to functional connectivity between brain areas dually engaged in autonomic and emotional regulation. For example, Schumann and colleagues examined resting‐state functional connectivity of the ventral medial prefrontal cortex with 260 independent regions‐of‐interest in a network‐based analysis conducted pre‐ and post‐an eight‐week HRV biofeedback intervention compared to a computer game control in 32 healthy, young men and women (Schumann et al. 2021). They observed increased connectivity between the ventral medial prefrontal cortex and the middle cingulate cortex, supplementary motor area, dorsal and ventral lateral prefrontal cortex, posterior and anterior insula, and right amygdala, suggesting a widespread effect of HRV biofeedback on the brain. Similarly, Nashiro and colleagues observed increased functional connectivity between the left amygdala and medial prefrontal cortex, and within canonical resting‐state networks implicated in emotion, in 98 healthy young adults who completed 5 weeks of a standard HRV biofeedback regimen (e.g., Lehrer et al. 2013) compared to a biofeedback regimen designed to reduce HR oscillations (Nashiro et al. 2023). These studies have begun to map chronic connectivity changes in CAN brain regions that share circuitry with emotion regulation networks both at rest and in task‐positive conditions.

The present study extends previous research by examining in‐the‐moment changes in functional connectivity during resonance paced breathing between 13 a priori selected brain regions of the CAN. Our delineation of the brain regions comprising the CAN was based on Beissner et al.'s activation likelihood estimation meta‐analysis of 43 neuroimaging experiments that assessed central processing of autonomic function in humans (Beissner et al. 2013). Their study yielded support for cortical and subcortical brain areas involved in autonomic processing that showed strong overlap with CAN regions identified in preclinical studies: middle cingulate cortex, thalamus, left amygdala, right amygdala, left anterior insula, right anterior insula, ventromedial prefrontal cortex, pregenual anterior cingulate cortex, right angular gyrus, ventral posterior cingulate cortex, left posterior insula, and right frontal insular cortex. As well, the nucleus of the solitary tract (Priovoulos et al. 2019) was included as the entry point of afferent cardiovascular signaling. We tested whether and how functional connectivity within this brain network changed during a brief episode of RPB compared to a brief episode of natural breathing.

Based on evidence that activation of the baroreflex instigates cardiovascular system communication with the brain and contributes to the brain's modulation of autonomic nervous system processes (Benarroch 1997; Goldstein 2001), we hypothesized increases in functional connectivity between CAN regions during a five‐minute episode of resonance paced breathing, compared to connectivity during natural breathing. Predictions about specific patterns of connectivity increases were not made due to the lack of previous research about the acute neural effects of slow, paced breathing interventions. Finally, we conducted exploratory analyses of selected demographic and psychosocial measures to gauge whether any observed connectivity changes from natural to resonance paced breathing systematically varied with age, sex, affective states, and perceived stress.

## 2 Method

This study combined neuroimaging and self‐report data from four different studies that included a functional magnetic resonance imaging (fMRI) arm with equivalent natural breathing and resonance paced breathing tasks. Each study was conducted at the same imaging research center using the same scanner and identical scan acquisition parameters. Though distinct, the four studies shared a common goal of understanding how breathing paced at six breaths‐per‐minute affects brain function.

### 2.1 Participants

Participants for this study included 177 individuals who took part in one of the four parent fMRI studies (55% women; 22.20 ± 5.17 years old). They were recruited from a large Northeastern US university and the communities surrounding it. One study was a randomized clinical trial that enrolled community women in outpatient treatment for substance use disorders (Price, Bates, Morgano, et al. 2022; Price, Bates, Pawlak, et al. 2022). The other three studies recruited primarily college students, some of whom met criteria for a depressive or substance use disorder (Bates et al. 2019; Lesnewich et al. 2019). Serious mental illness, pregnancy, and contraindications to MRI were common exclusion criteria across the studies. All participants provided written informed consent and were compensated for their time. This study's protocol was approved by the University's Institutional Review Board for the Protection of Human Subjects Involved in Research.

### 2.2 Materials and Measures

The *Perceived Stress Scale* (PSS) is a 10‐item self‐report measure of how often situations over the past month were experienced as stressful (Cohen et al. 1983). Items are scored on a 5‐point Likert‐type scale ranging from 0 (never) to 4 (very often). Higher scores indicated greater perceived stress. The Positive and Negative Affect Schedule (PANAS) is a 20‐item self‐report measure of current positive (10 items) and negative (10 items) affective (i.e., emotional) state (Watson et al. 1988). Items are scored on a 5‐point Likert‐type scale ranging from 1 (not at all) to 5 (very much). Higher scores indicate more positive or more negative affect, respectively. The *Beck Depression Inventory‐II* (BDI‐II) is a 21‐item self‐report measure of depression symptom severity over the past 2 weeks (Beck et al. 1996). Items are scored on a 4‐point Likert‐type scale ranging from 0 to 3 with anchors unique to each item. Higher scores indicate greater depression symptom severity. The *Beck Anxiety Inventory* (BAI) is a 21‐item self‐report measure of anxiety symptom severity over the past week (Beck et al. 1988). Items are scored on a 4‐point Likert‐type scale ranging from 0 (not at all) to 3 (severely), and higher scores indicate greater anxiety symptom severity.

### 2.3 Procedures

Following informed consent, participants completed self‐report questionnaires. For the RPB task, we standardized the paced breathing rate to six breaths‐per‐minute across participants, which produces a peak HRV amplitude in the spectral density function in a narrow range of frequencies around 0.1 Hz (~0.75–0.12, Vaschillo et al. 2006). Although there are some individual differences in resonance frequencies (Shaffer and Meehan 2020), a six breaths‐per‐minute pace approximates resonance sufficiently to engage the baroreflex, induce large cardiac oscillations, and promote adaptive physiology and emotional functioning (Russo et al. 2017; Song and Lehrer 2003; Steffen et al. 2017, 2021), without necessitating multiple testing sessions and extended protocols. Prior to entering the scanner, participants were taught to breath in synchrony with a visual pacer (E‐Z Air, Thought Technology Ltd., Plattsburgh, NY) set at the rate of one complete inhalation/exhalation breathing cycle every 10 s (i.e., 0.1 Hz frequency, six breaths‐per‐minute, Udo et al. 2013). There was an equal (5 s) duration for inhalation and exhalation with no pause between. Participants were instructed to inhale as the pacer went up and exhale as the pacer went down. They were taught to breathe slowly, but not too deeply, in a comfortable manner, to avoid hyperventilation. Task training lasted approximately 3 min. Participants were then affixed with several psychophysiological sensors as part of the larger parent studies, including electrocardiogram, a respiration belt, and in some studies, a finger pulsometer. In the scanner, participants viewed a projected screen positioned at the rear of the scanner bore and seen through a mirror attached to the head coil. During the natural breathing task, a typical resting‐state paradigm (Biswal et al. 1995; Lee et al. 2013), participants were instructed to look at a white fixation‐cross overlaid on a black background for 6 min. During the RPB task, the visual breathing pacer was projected onto the screen. The participants were instructed to inhale when the pacer went up and to exhale when the pacer went down during a five‐minute period. Compliance with the breathing task was verified via observation of the respiratory signal in real time, as well as during physiology data post‐processing. The results of paired *t*‐tests supported that the RPB task had the expected effect on cardiovascular/baroreflex function (LF HRV ms^2^ = 1051.74 ± 1231.39 during natural breathing and 4451.25 ± 3944.14 during RPB, *p* < 0.0001; Total power ms^2^ = 2377.86 ± 2251.09 during natural breathing and 5637.15 ± 4756.89 during RPB, *p* < 0.0001).

### 2.4 Neuroimaging Parameters

Imaging data were collected using a 3 T Siemens Trio scanner and 12‐channel head coil. Standard localizer, anatomical, scout, and field map scans were collected. High‐resolution anatomical images were acquired using a T1‐weighted MPRAGE protocol with parameters: repetition time (TR) = 1900 ms, echo time (TE) = 2.51 ms, matrix = 256 × 256 voxels, field‐of‐view (FOV) = 256 mm, voxel size = 1 × 1 × 1 mm, 176 1‐mm sagittal slices (0.5 mm gap). Functional blood‐oxygen‐level‐dependent (BOLD) data were acquired using single‐shot gradient echo‐planar imaging (EPI) sequences with parameters: TR = 2000 ms, TE = 25 ms, flip angle = 90°, matrix = 64 × 64 voxels, FOV = 192 mm, voxel size = 3 × 3 × 3 mm, 35 contiguous 3‐mm sagittal slices (1 mm gap). Respiration data were collected using a MRI‐compatible BIOPAC acquisition system (Biopac Systems, Goleta, CA) as part of the larger study. For the five‐minute RPB task, a total of 150 volumes were acquired; for the six‐minute natural breathing task, 180 volumes were acquired.

### 2.5 fMRI Pre‐Processing

AFNI 20.1.12 (Cox 1996) was used to analyze the fMRI data. All fMRI processing was performed using afni_proc. (https://afni.nimh.nih.gov/pub/dist/doc/program_help/afni_proc.py.html).

In order to directly compare connectivity between the two conditions, only first 150 volumes were used for analysis in the natural breathing task. Pre‐processing of the fMRI scans included removal of the first five time‐points, calculating outlier time points (defined as individual time‐points with more than 5% of within‐brain voxels flagged as outliers), de‐spiking, and spatial smoothing with 4 mm FWHM kernel. Each of the fMRI datasets was aligned to the base volume (with the lowest amount of outlier voxels) to correct for within‐scan head motion. Following motion correction, each fMRI image was co‐registered with a subject‐specific anatomical image and MNI standard template. GLM based linear regression was utilized to reduce the effect of motion and physiological noise on the fMRI signal. For both the RPB and the natural breathing tasks, the regression model included de‐meaned motion parameters, motion parameter derivatives, and scrubbing regressors for outlier time points, as well as for timepoints with more than 0.5 mm framewise displacement (Satterthwaite et al. 2013). To perform consistent preprocessing across both the tasks, we also included the 5 principal components from cerebral spinal fluid (CSF) and local white‐matter (WM) regression derived through ANATICO R. Both the CSF and WM masks were derived from Freesurfer segmentation. Following regression, each of the voxel time series was scaled to have a mean of 100 (range = 0 to 200). fMRI data from both tasks were bandpass filtered between 0.01 and 0.15 Hz to focus on low‐frequency fluctuations (0.01–0.1 Hz) as well as account for individual variability in resonance frequency across subjects (Vaschillo et al. 2006).

For each of the participants and scans, we calculated average frame‐wise displacement and number of individual time‐points considered outliers using 3dToutcount in AFNI. Briefly, 3dToutcount uses MAD (mean absolute deviation) to identify number of outlier voxels for each of the timepoints. When 5% or more of voxels are identified as outliers, the individual timepoint is flagged as an outlier. For the natural breathing task, mean and standard deviation of censor fraction (ratio of number of outlier timepoints to total timepoints) were 0.02 ± 0.08 and for the RPB task, the mean and standard deviation were 0.08 ± 0.15. There was a significant difference in the number of outlier points where the RPB task showed censor fraction compared to the normal breathing task (*p* < 0.005). Data from any participant where average framewise displacement was greater than 0.5 mm or when more than 20% of time‐points were considered outliers in the paced breathing or natural breathing task were excluded from further analysis. A total of 147 subjects passed the strict motion threshold and were included in the analysis.

### 2.6 Connectivity Analyses

To investigate functional connectivity differences between the RPB and natural breathing tasks, we implemented a region‐of‐interest (ROI) based approach. A total of 12 ROI coordinates from the CAN were defined based on Beissner and colleagues' meta‐analysis (Beissner et al. 2013) and a 6 mm sphere mask was created around the center coordinates in MNI space. The nucleus of the solitary tract (NTS) ROI mask was obtained from Priovoulos and colleagues (Priovoulos et al. 2019). We created a 6 mm sphere around the center of the mass voxel coordinates defined by Priovoulos et al. (2019) by creating a left and right NTS ROI. The two ROIs then were merged to create a single NTS ROI. These 13 ROI masks were used to extract pre‐processed BOLD fMRI time‐series for both the RPB and natural breathing tasks.

The functional connectivity between each of the ROIs (i.e., ROI pairs) was estimated by performing pair‐wise Pearson's correlations between the BOLD fMRI time‐series, resulting in a 13 × 13 correlation matrix for each of the participants and breathing tasks (RPB, natural breathing). These correlation coefficients were converted into Fisher's *z*‐scores for statistical comparison.

### 2.7 Statistical Analyses

All analyses used linear mixed modeling with functional connectivity of the 78 ROI pairs specified as the outcome variables and breathing task (RPB vs. natural breathing) specified as a main effect predictor. Linear mixed models were conducted separately for each ROI pair. Subject was specified as a nesting variable with the intercept designated as a random effect. Maximum likelihood estimation was used to fit each model. In order to control for inflated Type 1 error rate (i.e., multiple comparisons), an adaptive false discovery rate (FDR) (Benjamini and Hochberg 2000; Benjamini et al. 2006) correction was used on the *p*‐values associated with each functional connectivity value across the ROI pairs. The adaptive FDR was implemented using SAS PROC MULTTEST. Results were considered statistically significant when FDR *p* < 0.05.

The data were analyzed in several steps. *Step 1*: An initial set of linear mixed models was conducted to test any differential effect of pooling data together from the four studies by including Study as a main effect covariate in the models. *Step 2*: Next, we conducted another set of linear mixed models to examine the main effects of Breathing Task (natural breathing vs. RPB) on the ROI pairs. *Step 3*: Nonparametric Spearman rho correlations were computed between functional connectivity scores and the motion covariate (i.e., average framewise displacement per subject per scan) for each breathing task separately across all ROI pairs that showed a significant effect of Breathing Task. This was done to evaluate whether differential movement during RPB compared to natural breathing may have contributed to connectivity differences between breathing tasks. Low correlations would be indicative of negligible motion effects on the outcome variable of functional connectivity. Fisher's *z*‐test was used examine the significance of differences between correlations during natural breathing versus RPB tasks. We note that this analysis strategy was employed as an alternative to the inclusion of a motion covariate in Step 2 because the motion covariate was highly colinear with the breathing task manipulation and including both in the same model would have degraded the regression solution in this study (Tabachnick and Fidell 2019). *Step 4*: Lastly, to determine whether any observed differences between the breathing tasks were attributed to demographic and affective variables, we conducted linear mixed models that included the following covariates in interaction terms with the Breathing Task variable. To reduce multiple testing, four subsets of covariate models were specified: (1) age and sex, (2) perceived stress (PSS), (3) positive and negative affect (PANAS), and (4) depression (BDI‐II) and anxiety (BAI) symptoms. To further reduce multiple comparisons, the Step 4 models were conducted only for the ROI pairs that showed a significant effect of Breathing Task in Step 2. The covariate analysis results were FDR corrected within each set of results for each parameter.

## 3 Results

### 3.1 Main Effects of Breathing Task on Functional Connectivity

**FIGURE 1:**
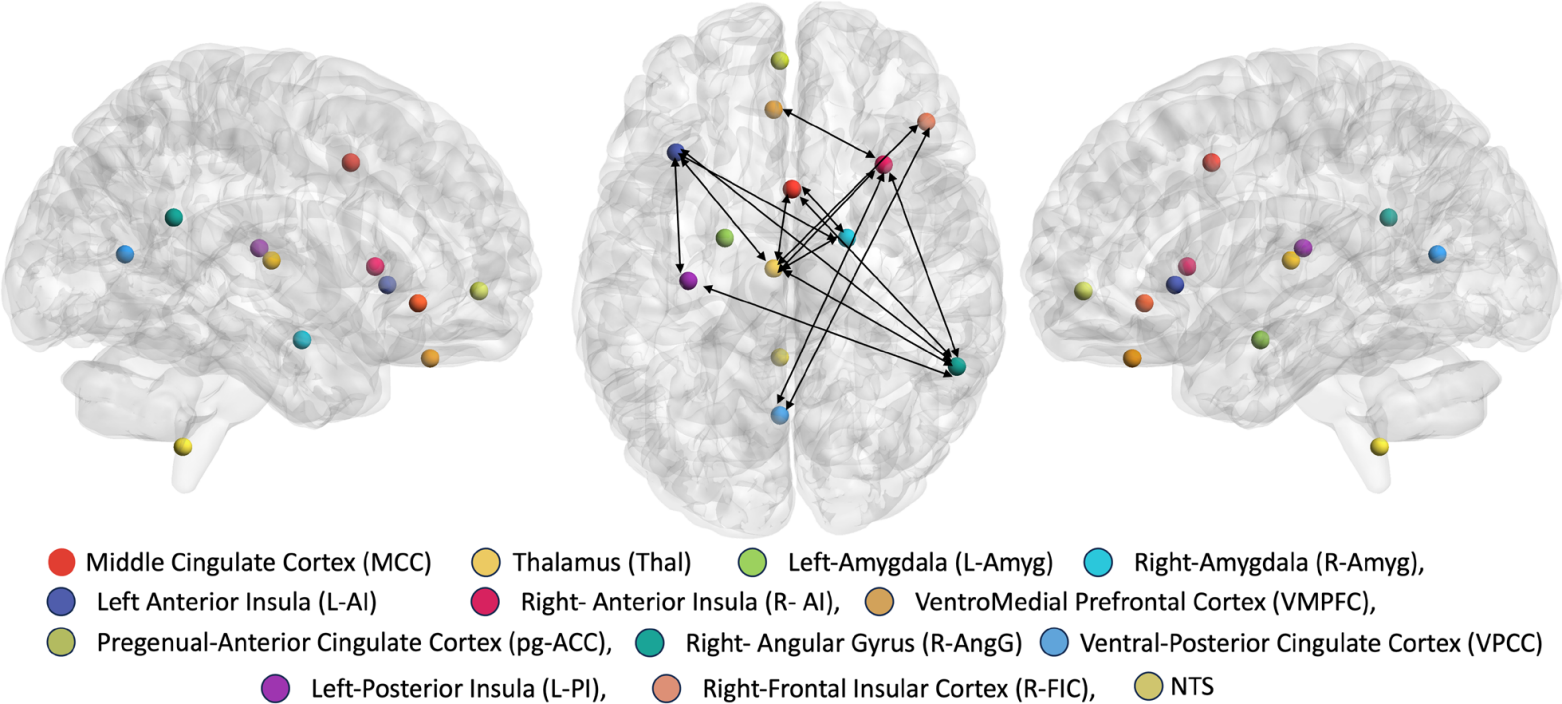
The central autonomic network (CAN) and region‐of‐interest (ROI) pairs with significantly different connectivity during resonance paced breathing (RPB) relative to natural breathing. The locations of 13 CAN ROIs are depicted as colored spheres superimposed on a glass‐brain surface shown in right and left sagittal and axial views. Double‐sided black arrows represent ROI pairs that demonstrated a significant main effect of Breathing Task in the linear mixed models; all significant ROI pairs demonstrated increased connectivity during RPB relative to natural breathing.

Figure 1 shows the 13 different CAN regions of interest on a glass‐brain surface created using Brain Net Viewer (Xia et al. 2013). In the initial set of linear mixed models, the main effect covariate of Study (the four parent studies that contributed data to the current study) was not statistically significant in any model. This indicated that differences between studies in methods and samples were not systematically related to CAN connectivity. In the second set of linear mixed models, following adaptive FDR correction for multiple comparisons, we observed a significant main effect of Breathing Task across 15 different ROI pairs (double headed arrows in Figure 1). Significantly increased functional connectivity was found between thalamus and left/right anterior insula, right angular gyrus, right frontal insular cortex, middle cingulate cortex, and right amygdala during the RPB task compared to the natural breathing task. We also observed an increase in functional connectivity during the RPB task between right angular gyrus and left/right anterior insula, middle cingulate cortex, and left posterior insula. Finally, there was a significant increase in functional connectivity of right amygdala with left anterior insula and middle cingulate cortex, between left anterior insula and left posterior insula, between right anterior insula and ventromedial prefrontal cortex, and between ventral posterior cingulate cortex and right‐frontal insular cortex during the RPB task, relative to the natural breathing task. These results are reported in Table 1.

**TABLE 1:** Results of linear mixed models of ROI pairs: Significant effects of breathing task on functional connectivity.

ROI pair	Estimates	*p*		Least square means	
	Breathing task	Un‐adjusted	Adaptive FDR‐corrected	Natural breathing	RPB
Thal‐RAngGyr	0.098	0.001	0.000	0.107	0.205
Thal‐RFIC	0.087	0.001	0.001	0.140	0.226
Thal‐LAI	0.075	0.001	0.002	0.167	0.242
LAI‐RAngGyr	0.068	0.001	0.003	0.112	0.180
RAngGyr‐LPI	0.063	0.001	0.003	0.080	0.143
MCC‐RAngGyr	0.069	0.001	0.003	0.141	0.210
MCC‐Thal	0.084	0.001	0.004	0.357	0.440
RAI‐RAngGyr	0.060	0.001	0.006	0.093	0.152
MCC‐RAmyg	0.059	0.001	0.008	0.082	0.140
Thal‐RAI	0.057	0.003	0.017	0.126	0.183
VentPCC‐RFIC	0.051	0.005	0.030	0.091	0.142
RAI‐VentPCC	0.051	0.008	0.041	0.073	0.124
Thal‐RAmyg	0.056	0.009	0.043	0.143	0.199
RAmyg‐LAI	0.043	0.010	0.043	−0.018	0.026
LAI‐LPI	0.046	0.012	0.048	0.231	0.277

*Note:* The 15 models out of 78 that showed a significant effect of breathing task following FDR correction are shown. Breathing Task is the estimate of the natural breathing versus RPB parameter. Connectivity between ROI pairs did not vary significantly across the four studies from which the data were drawn.

Abbreviations: FDR, False Discovery Rate; LAI, left anterior insula; LPI, left posterior insula; MCC, middle cingulate cortex; RAI, right anterior insula; RAmyg, right amygdala; RAngGyr, right angular gyrus; RFIC, right frontal insular cortex; RPB, resonance paced breathing; Thal, thalamus; VentPCC, ventromedial prefrontal cortex.

### 3.2 Motion Check

To determine whether any head motion that may have been introduced by the paced‐breathing task differentially influenced connectivity estimates in the RPB compared to the natural breathing task, we performed correlations between the functional connectivity and movement scores for the 15 significant ROI pairs. As shown in Table 2, Spearman's correlations between motion and connectivity scores were low in magnitude (0.00–0.25) across the 15 ROI pairs in both breathing tasks. Fisher's *z* tests without correction for multiple tests indicated one statistically significant difference between the RPB and natural breathing tasks in the relation of head movement to the left anterior insula‐right anterior gyrus connectivity scores. Overall, these results decrease the likelihood that differences in connectivity observed between breathing tasks reflected differences in head movement.

**TABLE 2:** Spearman's *ρ* (nonparametric) correlations of functional connectivity with motion scores.

ROI pair	Natural breathing		RPB		Fisher's *z*‐test for difference between correlations			
	ρ	*p*	ρ	*p*	*z*_1_	*z*_2_	*z*_Difference_	*p*
LAI‐LPI	0.19	0.02	0.20	0.01	0.19	0.20	−0.14	0.89
LAI‐RAngGyr	0.00	0.96	0.23	0.00	0.00	0.24	−1.99	0.05
MCC‐RAmyg	0.18	0.03	0.18	0.02	0.19	0.19	−0.01	0.99
MCC‐RAngGyr	0.04	0.65	0.07	0.39	0.04	0.07	−0.29	0.77
MCC‐Thal	0.06	0.48	0.03	0.76	0.06	0.03	0.27	0.78
RAI‐RAngGyr	0.12	0.14	0.15	0.07	0.12	0.15	−0.22	0.83
RAI‐VentPCC	0.25	0.00	0.11	0.20	0.25	0.11	1.24	0.22
RAmyg‐LAI	0.10	0.22	0.17	0.04	0.10	0.17	−0.61	0.54
RAngGyr‐LPI	0.13	0.11	0.20	0.02	0.13	0.20	−0.55	0.59
Thal‐LAI	0.03	0.68	0.11	0.20	0.03	0.11	−0.61	0.54
Thal‐RAI	0.11	0.17	0.15	0.07	0.11	0.15	−0.30	0.76
Thal‐RAmyg	0.05	0.51	0.08	0.34	0.05	0.08	−0.21	0.83
Thal‐RAngGyr	0.06	0.44	0.19	0.02	0.06	0.19	−1.08	0.28
Thal‐RFIC	0.08	0.31	0.21	0.01	0.08	0.22	−1.12	0.26
VentPCC‐RFIC	0.12	0.15	0.09	0.28	0.12	0.09	0.27	0.79

*Note:* *N* = 147. Variance for all ROI pairs = 0.014.

Abbreviations: LAI, left anterior insula; LPI, left posterior insula; MCC, middle cingulate cortex; RAI, right anterior insula; RAmyg, right amygdala; RAngGyr, right angular gyrus; RFIC, right frontal insular cortex; RPB, resonance paced breathing; Thal, thalamus; VentPCC, ventromedial prefrontal cortex.

### 3.3 Demographic and Psychological Covariate Analyses

No interaction effects between Breathing Task and age, sex, perceived stress, positive and negative affect, anxiety, or depression on connectivity were statistically significant following FDR correction.

## 4 Discussion

The major finding of this study was that compared to natural breathing, breathing paced at the 0.1 Hz resonance frequency of the arterial baroreflex was accompanied by increased functional connectivity between multiple brain regions of the central autonomic network involved in arousal states, interoception, emotional regulation, and cognitive control. The pattern of increased connectivity indicated that major relay and connector hubs with ascending and descending projections to brainstem, midbrain, and forebrain areas were affected by RPB. This acute modulation of connectivity during RPB supports the potential for volitional, in‐the‐moment modulation of affective and visceral states through respiratory control.

Of the 15 ROI pairs demonstrating significantly increased connectivity during resonance breathing, it is noteworthy that 10 pairs involved a subdivision of the insula. The insular cortex is a primary viscerosensory area that contributes to motivated behavior through its role in the representation of bodily states and urges (Bates and Buckman 2013). It is believed to underlie higher‐order interoception (Quadt et al. 2018) and the integration of interoceptive and exteroceptive information (Craig 2002; Critchley et al. 2004) with cognitive and emotional processes to guide motivated behavior (Namkung et al. 2017). We observed that during resonance paced breathing, anterior insula connectivity with posterior insula was increased, potentially suggesting more highly coordinated transfer of ascending interoceptive information. Connectivity between the right fronto‐insular cortex and both thalamus and ventral posterior cingulate cortex was increased during RPB. These findings support the idea that the insular cortex participates in the integration of afferent viscerosensory information with cognitive information relayed from higher cortical centers whose activity varies with arousal (Leech and Sharp 2014). It is thought that the insula's extensive connectivity to other brain regions and networks may point to its participation as a “gatekeeper” to executive control (Molnar‐Szakacs and Uddin 2022). During resonance paced breathing, bilateral anterior insula also showed increased connectivity with right angular gyrus, another brain region thought to integrate converging sensory information to support goal directed information processing and action (Seghier 2013). Right anterior insula functional connectivity with ventromedial prefrontal cortex was increased, pointing to a potential role for RPB in the context of emotional processing and risky decision making (Clark et al. 2008). Lastly, RPB increased left anterior insula connectivity with right amygdala, in line with evidence that arousal modulates effective connectivity between amygdala and insula (Wang et al. 2023).

Overall, the thalamus was involved in six of the 15 connectivity pairs that increased during RPB relative to natural breathing, consistent with its role as an important connector in the brain's network organization (Hwang et al. 2017; Shine et al. 2023). Limbic regions including the bilateral anterior insula and right amygdala showed increased connectivity with the thalamus, which receives information from the NTS, the first autonomic nucleus that receives viscerosensory afferent input from the body (Bates and Buckman 2013; Molnar‐Szakacs and Uddin 2022). Given the extensive connectivity of the thalamus to the cerebral cortex (Schiff 2008), its participation in the CAN is consistent with the idea that the thalamus plays an important role in the integration of afferent cardiac signals across functional brain networks, such as those involved in the regulation of arousal and emotion.

The right angular gyrus also showed increased connectivity with bilateral anterior insula, left posterior insula, thalamus, and mid‐cingulate cortex during RPB. Beissner et al. previously noted that the angular gyrus needs more study to understand its role in autonomic regulation (Beissner et al. 2013). In addition to its role in the CAN, the angular gyrus is involved in the frontoparietal control network, the default mode network, the ventral attention network, and others (Wei et al. 2019), highlighting its integrative role in sensory and cognitive information processing (Seghier 2013).

### 4.1 Clinical Implications

Understanding in‐the‐moment effects of brief respiratory interventions may contribute to the identification of biomarkers that can be used to increase the precision of matching individuals to HRV biofeedback and RPB interventions. From a precision medicine perspective, it is likely that respiratory interventions will be useful for many individuals, but not all. Using a computational physiological modeling approach, we previously showed that while a majority of persons exhibited positive changes in unobservable physiological functions during resonance paced breathing, a notable minority did not (Fonoberova et al. 2014). This was consistent with substantial literature showing that other interventions, such as psychopharmacology, cognitive behavioral therapy, and meditation are effective for some, but not all individuals with various bio‐behavioral disorders. It may be that increased connectivity during a brief episode of resonance breathing could provide a useful marker that predicts longer term benefits of HRV biofeedback and resonance breathing interventions.

Perhaps most importantly, in‐the‐moment effects of RBP may have clinical translation value (Bates et al. 2023). Existing studies of respiratory interventions such as HRV biofeedback and RPB have focused primarily on the positive long‐term effects of multi‐week courses of these interventions (Eddie et al. 2025). In addition to positive cumulative, chronic effects, the current pattern of results is consistent with the idea that respiratory interventions also may be candidate just‐in‐time interventions that individuals could employ to modulate physiological arousal. For example, women in outpatient addiction treatment who used brief episodes of RPB in response to affective triggers for alcohol and other drug use successfully mitigated increases in craving, compared to women randomized to sham breathing who experienced increased craving levels (Price, Bates, Morgano, et al. 2022; Price, Bates, Pawlak, et al. 2022). Goessl et al.'s (2017) meta‐analysis found that number of sessions (from 1 to 50) did not moderate treatment efficacy, supporting both immediate and long term benefits of HRV biofeedback and RPB interventions with respect to anxiety and stress reduction. More research is needed to uncover transdiagnostic mechanisms such as reward processing (Arinel and Adbdelaal 2025) and inhibitory control (Yan et al. 2022) that may underlie the link between RPB and neurobiological processes. We speculate that increased functional connectivity in the CAN may be a neural substrate of reduced symptoms of craving, anxiety, and stress. Thus, RPB may be an intervention that individuals could use intentionally in their daily lives to interrupt or mitigate negative affective states in real time.

### 4.2 Limitations

This study aggregated data from four distinct studies that sampled from both clinical and nonclinical populations. In addition, while the natural breathing condition occurred first in the imaging session, the RPB task was preceded by different cognitive or perceptual tasks across studies. Although we found that changes in connectivity during RPB did not differ significantly across the four studies, we cannot definitively rule out the contribution of task context or of sample heterogeneity to the results. Further, the current study did not find the connectivity of the NTS with other CAN regions to be different between the two breathing conditions. A potential limitation is the lower signal‐to‐noise ratio of brainstem regions in conventional 3 T fMRI (Brooks et al. 2013). In addition, the size of the current NTS ROI was larger than the anatomical dimensions of the NTS (Naidich et al. 2009) and likely encompassed other nuclei. See Figure S1 for the anatomically defined NTS size (Priovoulos et al. 2019) and center of mass coordinates. Connectivity of the NTS with larger structures in the CAN needs to be validated using higher resolution fMRI data. The RPB protocol included an element of “attention to breathing”, which a previous study suggested may contribute to increases in amygdala–prefrontal cortex functional connectivity during aversive emotional picture stimulation (Doll et al. 2016). The future examination of attention to breathing with and without breath pacing may inform shared and unique neural effects. A visual pacer was not used in the control task so as not to compromise individual differences in spontaneous breathing rates. We note that visual information as well as afferent viscerosensory information goes to the lateral geniculate nucleus of the thalamus, a region implicated in our primary findings. Indeed, all brain regions of the central autonomic network are highly multifunctional, rather than dedicated solely to autonomic information processing streams. Future studies that pace breathing using different sensory modalities, or that more finely discriminate different thalamic subregions and nuclei, are needed to isolate the role these regions play in autonomic processing. We performed several steps to reduce the effect of motion on the between‐task results. These included motion regression, “scrubbing”, and the removal of data from participants with movement over a predetermined threshold. However, given that the remaining motion estimates per subject were highly collinear with the breathing task variable, we could not include motion as a covariate in the models (Tabachnick and Fidell 2019). This multicollinearity may be due to the nature of the RPB paradigm. Although we have shown that there is little impact of motion on the observed breathing task differences for the ROI pairs investigated, future studies should validate these results using independent data sets. Relatedly, it should be noted that blood pulsation and respiration are potential sources of noise added to the neural signal, and in most cases it is advantageous to employ a pipeline that removes physiological noise from the fMRI signal (Birn et al. 2014, 2008). The RPB paradigm we used is an exception to this best practice as these physiological effects would be expected to be highly correlated with the neural signal changes instigated during RPB. It is likely that the preprocessing did not remove all mechanical physiological components from the BOLD signal. Thus, the possibility of residual mechanical physiological noise should be considered in interpreting the connectivity results. Finally, this investigation focused on the CAN and the results do not address the effect of RPB on brain regions outside this network.

### 4.3 Conclusions and Future Directions

The present results add to the growing literature on respiratory modulations of brain activity during natural breathing (Heck et al. 2022) by demonstrating that slow breathing paced at a resonance frequency of the arterial baroreflex simultaneously modulated the connectivity of brain areas involved central autonomic control, cognition, and emotion regulation. We note that there has been debate about the contribution of respiratory and cardiac signals to the BOLD signal and connectivity analysis (Birn et al. 2009). Earlier perspectives viewed autonomic processes and influences as purely “nuisance” variables and developed strategies to differentiate physiological “noise” from neural changes. Continued progress in understanding afferent cardiovascular and respiratory contributions to neural processes has qualified this perspective (e.g., Goheen et al. 2023), although much work remains to understand the different mechanisms of oscillation in the brain. Cardiorespiratory signals can modulate neural processes in the service of improved mental health, as underscored by cognitive and emotional effects of respiratory interventions such as RPB and HRV biofeedback.

An important task for future research is to discover the level and time scale of cardiorespiratory interventions' clinical effects. While the positive clinical effects of multi‐week biofeedback interventions are well established, more research is needed to understand the immediate effects of RPB on stress, anxiety, and other high‐risk states. Cardiorespiratory manipulations such as RPB have real‐time neural effects that potentially may be utilized for volitional, self‐initiated control of arousal and its integration with cognitive and emotional information processing.

## Author Contributions

**M. E. Bates:** conceptualization, supervision, funding acquisition, methodology, writing – original draft, writing – review and editing, project administration. **L. M. Lesnewich:** investigation, writing – original draft, writing – review and editing. **A. P. Pawlak:** formal analysis, visualization, writing – original draft, writing – review and editing. **J. F. Buckman:** funding acquisition, methodology, supervision. **S. Gohel:** formal analysis, methodology, visualization, writing – original draft, writing – review and editing.

## Funding

This research was supported in part by the National Institutes of Health grants (R01 AA023667, K24 AA021778, R21 AA022748, and R03 DA031060).

## Conflicts of Interest

The authors declare no conflicts of interest.

## Supporting information


**Figure S1:** Overlap between the anatomically defined size of NTS ROI based on the standard template as defined by Priovoulos et al. (2019) centers of mass (NTS –L: −3 44–50, NTS –R: 2 45–50; shown in Red) and the current study's NTS ROI created around these center of mass coordinates (shown in Yellow). L = left. NTS = nucleus of the solitary tract. R = right. ROI = region of interest.

## Data Availability

The data that support the findings of this study are available on reasonable request from the corresponding author.
