# Supplementary material for: Functional Connectivity Within the Central Autonomic Network Increases During Resonance Paced Breathing at 0.1 Hz
Source: Psychophysiology. 2026 Feb 23;63(2):e70263. doi: 10.1111/psyp.70263 (PMC12929929; doi:10.1111/psyp.70263)
Supplement: Supplementary file 1 — Figure S1: Overlap between the anatomically defined size of NTS ROI based on the standard template as defined by Priovoulos et al. (2019) centers of mass (NTS –L: −3 44–50, NTS –R: 2 45–50; shown in Red) and the current study's NTS ROI created around these center of mass coordinates (shown in Yellow). L = left. NTS = nucleus of the solitary tract. R = right. ROI = region of interest. [file PSYP-63-e70263-s001.docx]

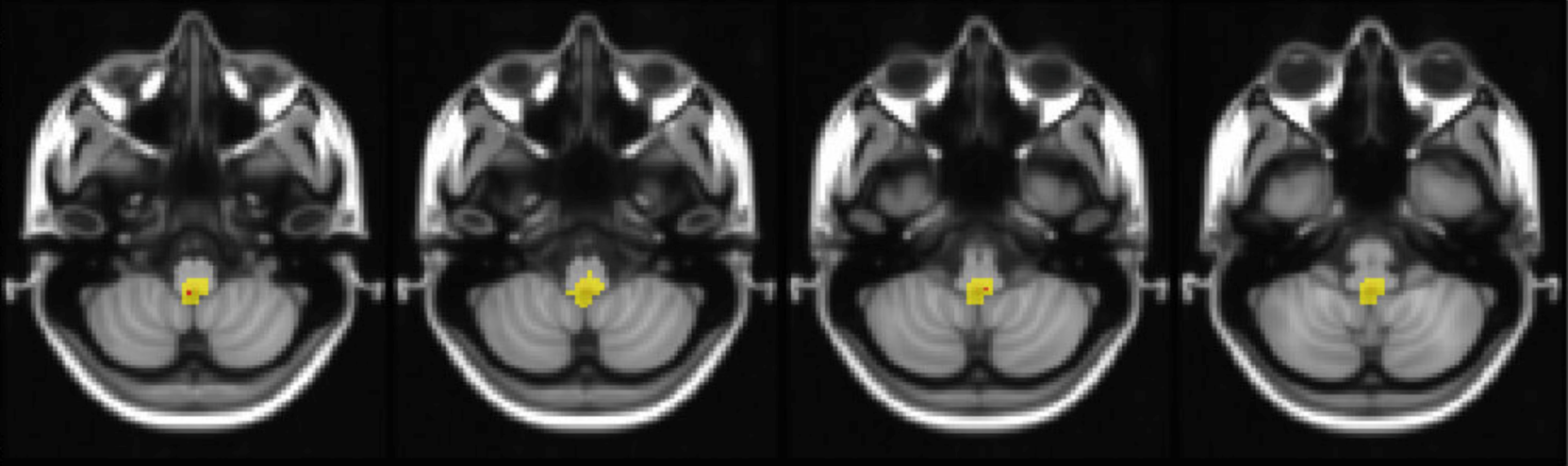
 Supplementary Figure 1: Overlap between the anatomically defined size of NTS ROI based on the standard template as defined by Priovoulos and colleagues’ (2019) centers of mass (NTS –L : -3 44 -50, NTS –R : 2 45 -50; shown in Red) and the current study’s NTS ROI created around these center of mass coordinates (shown in Yellow). L = left. NTS = nucleus of the solitary tract. R = right. ROI = region of interest.
